# Celiac Disease-Related Enamel Defects: A Systematic Review

**DOI:** 10.3390/jcm13051382

**Published:** 2024-02-28

**Authors:** Alessio Danilo Inchingolo, Gianna Dipalma, Fabio Viapiano, Anna Netti, Irene Ferrara, Anna Maria Ciocia, Antonio Mancini, Daniela Di Venere, Andrea Palermo, Angelo Michele Inchingolo, Francesco Inchingolo

**Affiliations:** 1 Department of Interdisciplinary Medicine, School of Medicine, University of Bari “Aldo Moro”, 70124 Bari, Italy; ad.inchingolo@libero.it (A.D.I.); giannadipalma@tiscali.it (G.D.); viapianofabio96@gmail.com (F.V.); annanetti@inwind.it (A.N.); ire.ferra3@gmail.com (I.F.); anna.ciocia1@gmail.com (A.M.C.); dr.antonio.mancini@gmail.com (A.M.); daniela.divenere@uniba.it (D.D.V.); angeloinchingolo@gmail.com (A.M.I.); 2 College of Medicine and Dentistry, Birmingham B4 6BN, UK; andrea.palermo2004@libero.it

**Keywords:** enamel, celiac disease, dental enamel defects, DED, molar incisor hypomineralization, MIH, oral health, early diagnosis, interdisciplinary healthcare, dental care

## Abstract

Introduction: This systematic review aims to elucidate the intricate correlation between celiac disease (CD) and dental enamel defects (DED), exploring pathophysiological mechanisms, oral health implications, and a dentist’s role in early diagnosis. Materials and methods: Following PRISMA guidelines, a comprehensive search from 1 January 2013 to 1 January 2024 across PubMed, Cochrane Library, Scopus, and Web of Science identified 153 publications. After exclusions, 18 studies met the inclusion criteria for qualitative analysis. Inclusion criteria involved study types (RCTs, RCCTs, case series), human participants, English language, and full-text available. Results: The search yielded 153 publications, with 18 studies meeting the inclusion criteria for qualitative analysis. Notable findings include a high prevalence of DED in CD patients, ranging from 50 to 94.1%. Symmetrical and chronological defects, according to Aine’s classification, were predominant, and significant associations were observed between CD severity and enamel defect extent. Conclusions: The early recognition of oral lesions, particularly through Aine’s classification, may signal potential CD even in the absence of gastrointestinal symptoms. Correlations between CD and dental health conditions like molar incisor hypomineralization (MIH) emphasize the dentist’s crucial role in early diagnosis. Collaboration between dentists and gastroenterologists is essential for effective monitoring and management. This review consolidates current knowledge, laying the groundwork for future research and promoting interdisciplinary collaboration for improved CD-related oral health outcomes. Further large-scale prospective research is recommended to deepen our understanding of these issues.

## 1. Introduction

**Figure 1:**
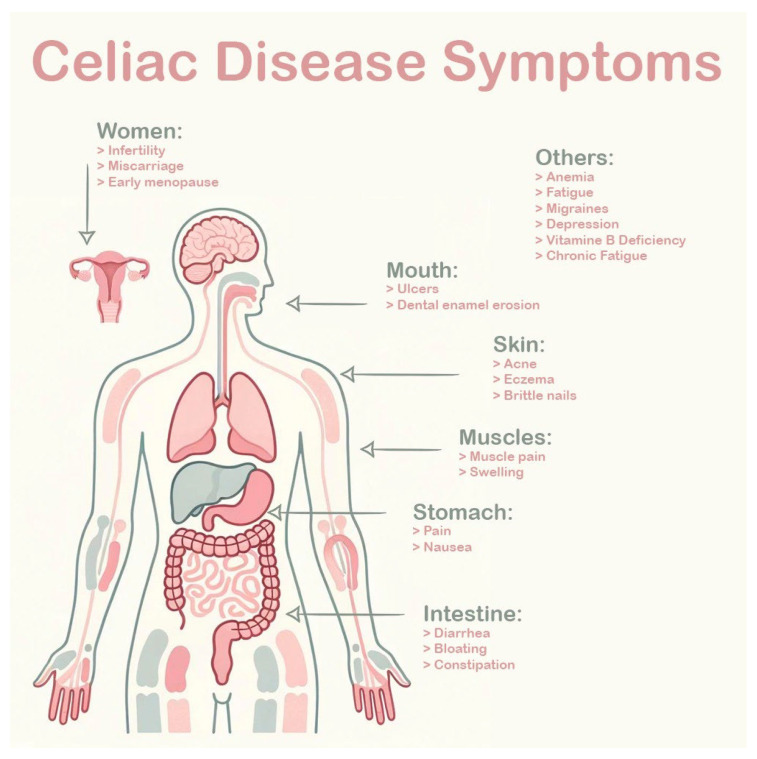
CD symptoms.

Celiac disease (CD), a multifaceted autoimmune disorder triggered by the consumption of gluten in genetically susceptible individuals, presents an intricate interplay of systemic and localized manifestations (Figure 1) [1]. This comprehensive exploration delves into the intricate correlation between celiac disease and dental enamel defects (DED), shedding light on pathophysiological mechanisms, oral health implications, and the critical role of dentists in early diagnosis [1]. As we navigate through the complexities of celiac disease and its impact on dental enamel, the importance of a systematic review becomes evident, aiming to consolidate existing knowledge, address diagnostic challenges, and empower dental practitioners in the identification of potential celiac patients [2]. 

**Figure 2:**
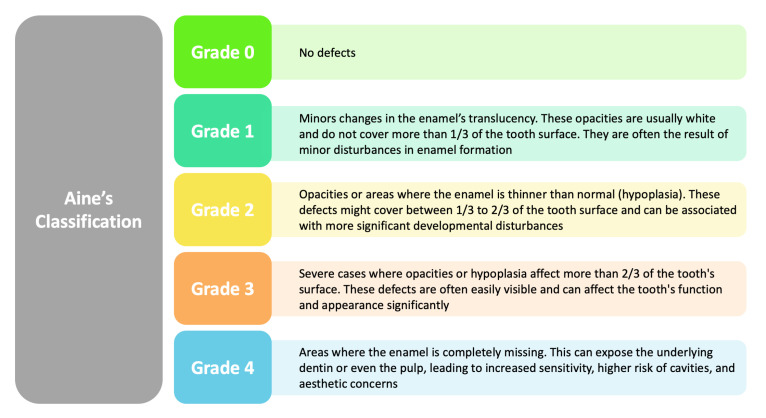
Aine’s classification: classification of systematic and chronological enamel defects.

**Figure 3:**
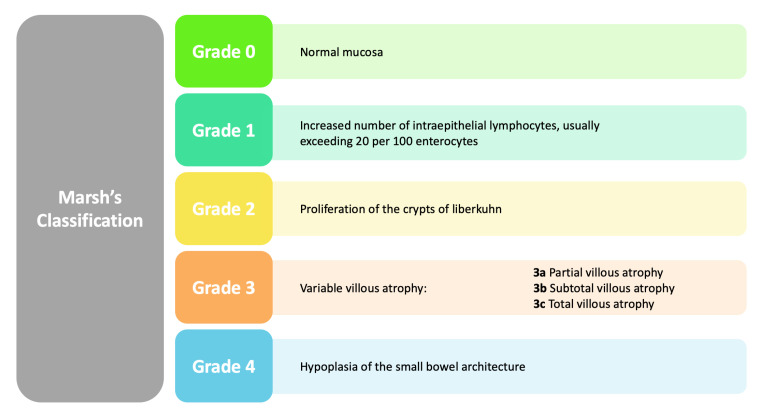
Marsh’s classification: histopathological classification of celiac disease.

In addition to considering the impact of celiac disease on dental enamel, this review incorporates classifications that further enrich our understanding of related defects [3]. Aine’s classification of enamel defects offers a structured approach to categorize the diverse manifestations observed, while Marsh’s classification of intestinal lesions in celiac disease provides additional context to the systemic implications [3,4]. Both classifications contribute valuable perspectives, reinforcing the need for a comprehensive examination of the intricate relationship between celiac disease and DED (Figure 2 and Figure 3). 

CD, also known as gluten-sensitive enteropathy, is an autoimmune disorder characterized by an aberrant immune response to gluten, a protein found in wheat, rye, and barley [1]. The prevalence of CD has seen a notable rise in recent years, affecting individuals of all ages, ethnicities, and geographic locations [5]. While genetic predisposition is a key factor, environmental triggers, such as viral infections and early exposure to gluten, contribute to the development of this intricate condition [6]. 

**Figure 4:**
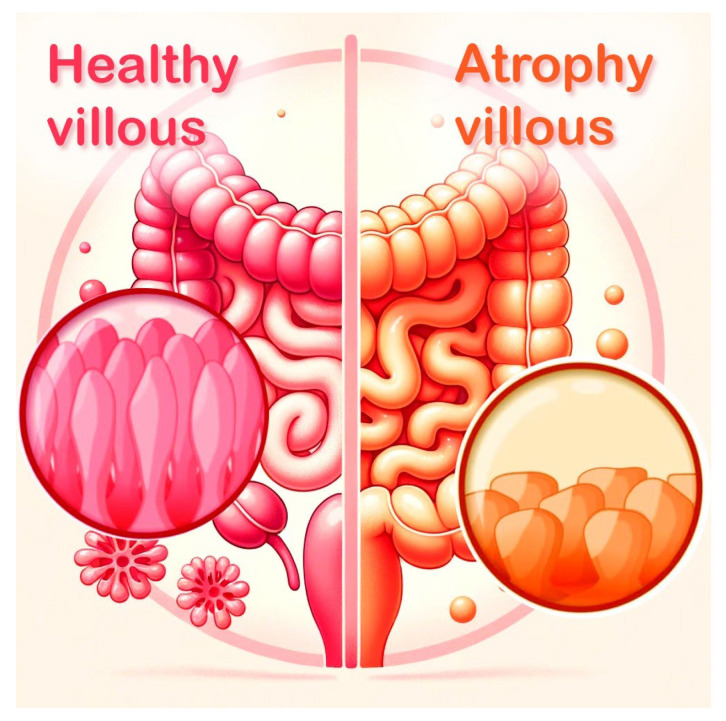
Well-distributed and ordered villous structures in a healthy subject; in a celiac patient, the villous structures are totally disappeared and flattened.

Understanding the pathophysiological mechanisms underlying CD is fundamental to discerning its diverse clinical manifestations [7]. Upon the ingestion of gluten by susceptible individuals, an aberrant immune reaction is initiated, leading to the inflammation and atrophy of the intestinal villi, which are crucial for nutrient absorption [8] (Figure 4). This results in the malabsorption of essential nutrients, manifesting clinically as diarrhea, weight loss, fatigue, anemia, and in children, failure to thrive. Celiac disease is genetically predisposed, linked to specific HLA-DQ2 and HLA-DQ8 haplotypes, and diagnosis typically involves serological testing for specific antibodies, such as anti-tissue transglutaminase and anti-endomysial antibodies, followed by confirmatory intestinal biopsy [8]. The cornerstone of management is a strict lifelong gluten-free diet, which can lead to the resolution of symptoms, normalization of antibody levels, and recuperation of the intestinal mucosa [9,10,11]. 

**Figure 5:**
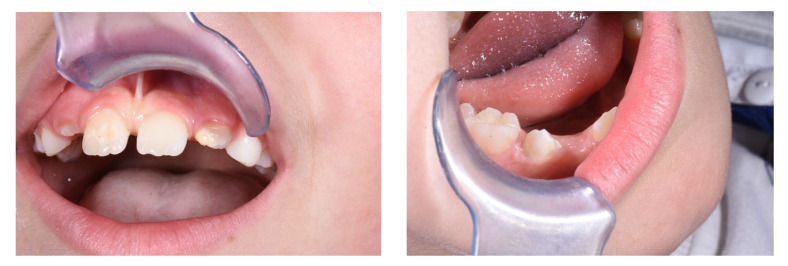
Example of DED in patient with CD.

The impact of celiac disease extends beyond the confines of the gastrointestinal tract, involving various extraintestinal manifestations, including oral health [12]. The oral cavity serves as a window to systemic health, and alterations in oral tissues can often signify underlying systemic conditions [13]. In the context of CD, oral manifestations are diverse and may include aphthous ulcers, recurrent oral ulceration, and DEDs. DEDs, in particular, emerge as a noteworthy aspect of CD-related oral manifestations [14] (Figure 5). Research suggests a direct influence of gluten on enamel development, with implications for its mineralization process [15]. Studies propose that CD may interfere with the normal mineralization of dental enamel, resulting in structural abnormalities [16]. Enamel defects, such as pitting, grooving, and discoloration, have been reported in individuals with CD, providing potential oral markers for the condition [17,18,19,20,21,22,23,24,25,26,27,28,29,30,31,32,33,34,35,36,37,38,39,40,41,42,43,44,45,46]. 

While DEDs can manifest in various conditions, distinguishing those associated with CD is crucial for accurate diagnosis and management. Celiac-related enamel defects may share similarities with other enamel pathologies, such as amelogenesis imperfecta or enamel hypomineralization, necessitating a meticulous approach to differential diagnosis. 

Establishing diagnostic criteria for celiac-related enamel defects involves a combination of clinical observation, patient history, and, when appropriate, serological and genetic testing [47]. Challenges arise due to the overlap of symptoms with other dental conditions and the variability in enamel defect presentations. The importance of a multidisciplinary approach, involving both gastroenterologists and dentists, becomes evident in navigating these diagnostic intricacies [48,49,50,51,52,53,54,55,56,57,58,59]. 

The significance of exploring the correlation between CD and DEDs lies in the potential for early diagnosis and management. DEDs can be an early indicator of CD, offering a window of opportunity for timely intervention and improved patient outcomes [22]. Moreover, understanding the nuances of this correlation can enhance the overall quality of care for individuals affected by CD [60,61,62,63]. 

The oral cavity is intricately connected to systemic health, and changes in oral tissues can reflect underlying systemic conditions [64]. Recognizing the oral manifestations of CD, particularly enamel defects, emphasizes the role of dentists as integral contributors to the comprehensive healthcare team [65]. A holistic approach to patient care involves considering oral health as a reflection of systemic well-being [66].

In light of the complexity surrounding CD, its oral manifestations, and the diagnostic challenges in DEDs, there arises a compelling need for a systematic review [67]. Such a review aims to synthesize existing evidence, elucidate patterns in celiac-related enamel defects, and establish guidelines for differential diagnosis [68]. A systematic approach ensures the thorough examination of the available literature, addressing any gaps in knowledge and providing a robust foundation for future research and clinical practice [69]. 

The proposed systematic review seeks to unravel the intricate relationship between CD and DEDs with a specific focus on the dentist’s role in early diagnosis [70]. Dentists, being at the forefront of oral health assessment, can act as sentinels in identifying potential cases of CD through the careful observation and evaluation of enamel defects [71]. This proactive approach can contribute to the timely referral of patients for further investigation and collaboration with gastroenterologists [72,73,74,75]. 

Through a meticulous examination of the existing literature, this systematic review aims to consolidate knowledge, provide insights into diagnostic intricacies, and empower dentists with valuable information for enhanced patient care [76]. The overarching goal is to bridge the gap between gastroenterology and dentistry, fostering collaboration for a more comprehensive and integrated approach to the diagnosis and management of CD [77]. 

The interplay between CD and DEDs is a multifaceted terrain that warrants in-depth exploration. This comprehensive review endeavors to unravel the complexities, emphasizing the interconnectedness of oral and systemic health. By recognizing the dentist’s pivotal role in early CD diagnosis, this systematic review aspires to contribute to a holistic understanding of the condition, ultimately improving patient outcomes and promoting interdisciplinary collaboration in healthcare.

## 2. Materials and Methods

### 2.1. Search Processing

The present systematic review adheres to the PRISMA and International Prospective Register of Systematic Review Registry guidelines (ID 501763) [78]. We conducted searches on PubMed, Cochrane Library, Scopus, and Web of Science to identify relevant papers within the timeframe from 1 January 2013 to 1 January 2024, with an English-language criterion. Our search strategy employed a combination of terms tailored to the investigation’s focus. Consequently, the Boolean keywords employed to identify papers written in the last 10 years were: (“celiac disease” OR “gluten sensitivity” OR “gluten enteropathy”) AND (“enamel defects” OR “dental enamel” OR “tooth enamel”).

### 2.2. Inclusion Criteria

Reviewers conducted a thorough assessment evaluating all eligible trials based on the following inclusion criteria: (1) randomized control trial or randomized controlled clinical trial, (2) case series comprising more than five clinical cases, (3) studies involving human participants, (4) full text-available, and (5) articles published in the English language. Conversely, exclusion criteria were established as follows: (1) systematic or literature reviews, (2) editorials, (3) case reports (those with only one case), (4) case series with fewer than five cases, (5) studies conducted in vitro, (6) studies involving animals, and (7) articles not published in the English language. These criteria were rigorously applied during the article selection process to ensure that the selected studies for the systematic review met the standards of quality and relevance.

### 2.3. Quality Assessment

The quality of the included papers was assessed by two reviewers, RF and EI, using the ROBINS tool developed to assess the risk of bias in the results of non-randomized studies that compare the health effects of two or more interventions. Seven points were evaluated, and each was assigned a degree of bias. A third reviewer (FI) was consulted in the event of a disagreement until an agreement was reached.

## 3. Results

**Figure 6:**
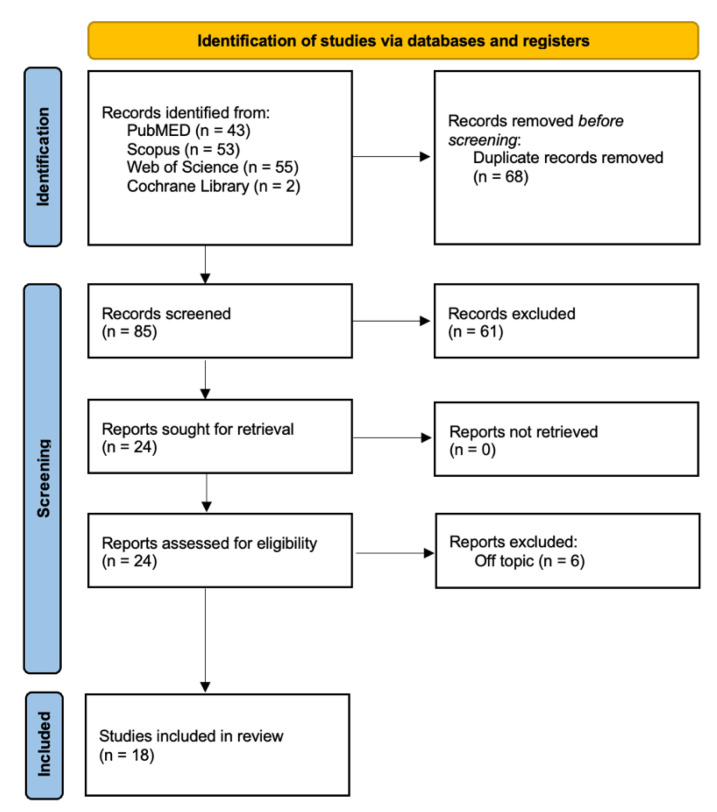
PRISMA flowchart of the inclusion process.

A comprehensive search across multiple databases, namely PubMed (43), Scopus (53), Cochrane Library (2), and Web of Science (55), yielded a total of 153 publications. Following the removal of duplicates (68), 85 unique articles remained. A meticulous examination of titles and abstracts led to the exclusion of 61 publications. Subsequently, the authors successfully retrieved and assessed the eligibility of the remaining 24 articles. During this process, 6 publications were excluded due to their lack of relevance to the topic. Ultimately, 18 studies were included in the review for qualitative analysis (Figure 6 and Table 1).

The studies analyzed examined the oral health implications of CD, revealing that CD patients often experience more severe dental and enamel issues compared to controls. Specifically, CD patients had higher DMFT scores, indicating greater dental decay, and a significant presence of DEDs, with 34.5% of patients exhibiting mostly mild enamel defects and a higher occurrence of aphthous stomatitis. A substantial 68.6% of CD patients reported symptoms of dry mouth. Children with CD showed a higher incidence of enamel defects and recurrent aphthous stomatitis than those without CD. Despite a higher incidence of caries in deciduous teeth, no increased prevalence of caries was noted in permanent teeth for the CD group. Additionally, CD was linked to a significantly higher chance of developing MIH and specific enamel defects, particularly in certain teeth. This study underscores the need for heightened dental care and monitoring in CD patients due to these associations.

### Quality Assessment and Risk of Bias

**Figure 7:**
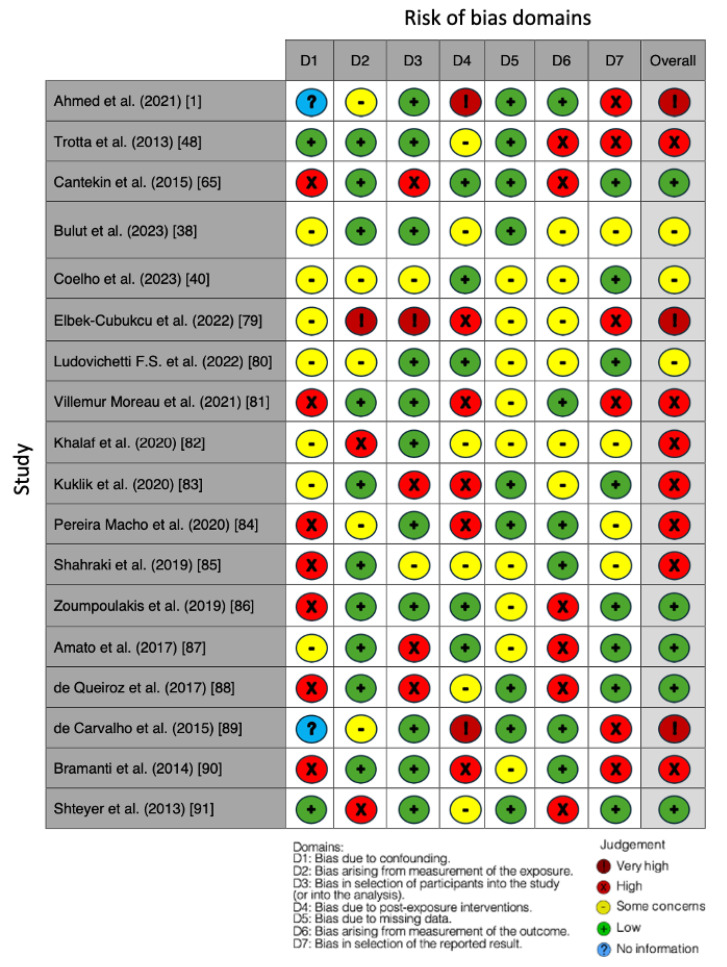
Bias assessment. Ahmed et al. (2021) [1]; Trotta et al. (2013) [48]; Cantekin et al. (2015) [65]; Bulut et al. (2023) [38]; Coelho et al. (2023) [40]; Elbek-Cubukcu et al. (2022) [79]; Ludovichetti F.S. et al. (2022) [80]; Villemur Moreau et al. (2021) [81]; Khalaf et al. (2020) [82]; Kuklik et al. (2020) [83]; Pereira Macho et al. (2020) [84]; Shahraki et al. (2019) [85]; Zoumpoulakis et al. (2019) [86]; Amato et al. (2017) [87]; de Queiroz et al. (2017) [88]; de Carvalho et al. (2015) [89]; Bramanti et al. (2014) [90]; Shteyer et al. (2013) [91].

The risk of bias in the included studies is reported in Figure 7. Bias resulting from confounding the majority of studies is a high-risk form of bias, while that arising from measurement parameters is a low-risk form of bias. Many studies have a low risk of bias due to bias in the selection of participants. Post-exposure bias cannot be calculated due to high heterogeneity. Bias due to missing data is low in many studies. Bias arising from the measurement of the outcome is low. Bias in the selection of the reported results is high in most studies. The final results show that seven studies have a high risk of bias, three have a very high risk of bias, and five have a low risk of bias. In three studies and in three domains, bias could not be established.

## 4. Discussion

The interest in CD has grown significantly in recent years, as it has become widely known that this systemic autoimmune disease of the small intestine affects a significant number of people worldwide. CD is caused by the ingestion of gluten-containing products in genetically predisposed individuals, requiring extensive testing for diagnosis, including sophisticated serologic tests such as TTG IgA antibodies, followed by a proximal intestinal biopsy, considered as the gold standard [5].

The prevalence of CD is increasing, affecting 40–60 million people worldwide and 6–8 million in India [1].

In the Western world, the prevalence of CD is high (1/150), characterized by severe complications that double the mortality rate [48].

Clinically, CD occurs in three different forms: classic, non-classic, and asymptomatic. The classic symptoms are gastrointestinal, such as diarrhea, weight loss, and anemia, but other organs are also affected, such as the skin, brain, liver, and teeth. The oral manifestations of CD include DED, which is common, with a prevalence of more than 80 percent [48].

The factors contributing to DEDs IIe Immune damage, nutritional disorders, and genetic factors. DEDs are considered specific if they are present symmetrically and chronologically on both sides and distributed in all four quadrants of the permanent teeth, with incisors and molars as the most commonly affected teeth [88]. 

DED is irreversible and does not regress with a gluten-free diet (GFD). Other differential diagnoses of DED include enamel hypoplasia, fluorosis, amelogenesis imperfecta, and localized trauma/infection. Several studies have shown a high incidence of DED in celiac patients, suggesting a correlation with the extent of intestinal lesions [1,48,85].

CD-induced calcium malabsorption is hypothesized to affect enamel formation, especially in early life. 

The study by Ahmed et al. [1] involves a sample of 140 Asian patients with CD, including untreated patients and those already on a GFD for at least 1 year. Forty subjects were included as a control group (CG). From the study, 94.1% of patients with CD had oral and/or dental manifestations. A total of 66.9% of patients with CD presented with DED, significantly higher than the CG (20%). About two-thirds of patients with CD exhibited DED [1].

Trotta et al. [48] conducted a study of 54 celiac patients of Caucasian ethnicity. A total of 85.2% of the patients reported DED, and grade 1 defects according to Aine’s classification [3] were the most common. Dental enamel defects (DEDs) appear to be more severe in patients with the classic form of CD [48]. 

In a study of 65 Iranian children [85] with CD and 60 children without the condition, DED and other oral manifestations were examined. In the group with CD, the prevalence of enamel defects was 50 percent compared with 12 percent in the CG. The classification of defects followed Aine’s criteria [3], with defects mainly grade I, but some cases had severe grade III and IV defects. The investigation revealed a significant correlation between the presence of CD and symmetrical DED [85]. Caries in deciduous teeth was higher in children with CD, but there was no higher prevalence of caries in permanent teeth, despite the fact that the group with CD had lower sugar consumption than the CG (25% vs. 47%). Xerostomia was significantly observed in more children with CD than in the CG (15.4% vs. 5%) [85]. 

Some researchers suggest a possible common genetic basis or the possibility that enamel defects are caused by nutritional deficiencies related to CD [88].

In a study of 45 Brazilian individuals with CD, Mussolino de Queiroz [88] shows a strong correlation between CD and GFD, with some significant associations between specific types of defects and the timing of the introduction of the GFD. Further genetic research is recommended for a better understanding of the relationship [88].

The relationship between CD and molar incisor hypomineralization (MIH) has been explored in several studies [79,83]. 

**Figure 8:**
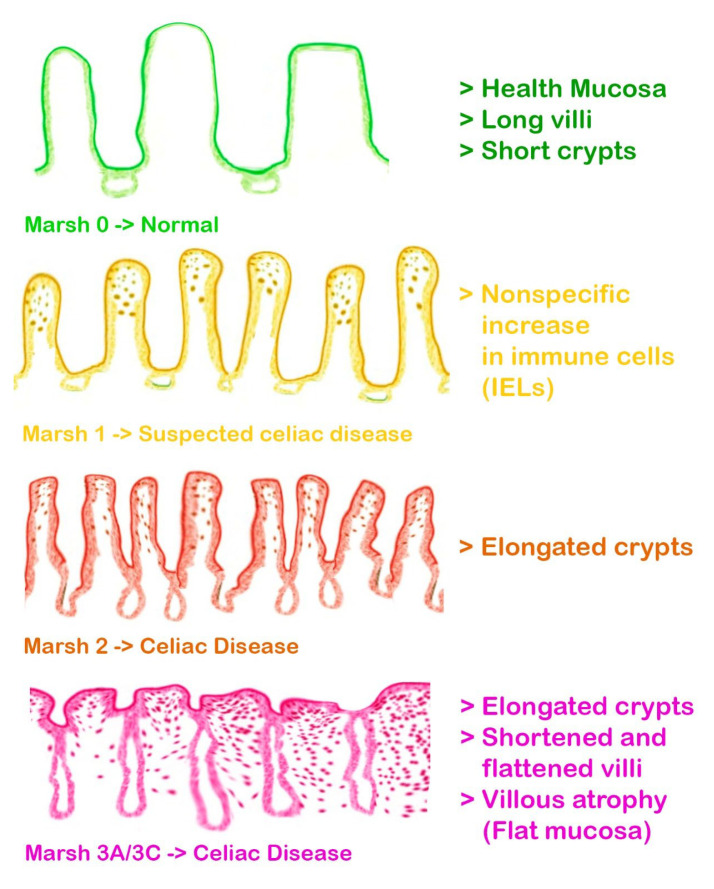
Marsh’s classification. (0) Normal mucosa; (1) Increased number of intraepithelial lymphocytes, usually exceeding 20 per 100 enterocytes; (2) Proliferation of the crypts of liberkuhn; (3) Variable villous atrophy, subdivided from A to C depending on the severity of the atrophy.

Cigdem Elbek-Cubukcu [79] studied 62 children with CD and 64 healthy children between January and August 2021. In the group of children with CD, there was a 61% prevalence of DED, mainly in the form of MIH, compared with 65.6% of healthy children in the CG who did not have MIH [79]. In addition, a significant inverse relationship was identified between the number of MIHs and the age at diagnosis of CD, indicating that early diagnosis might be related to a lower incidence of MIH [79]. Regarding dental caries experiences, there were no significant differences between the groups, but it was found that the number of caries in permanent teeth (DMFT) was correlated with the different Marsh types (Figure 8), with the Marsh 2 group having a higher number of caries. In addition, reduced saliva buffering capacity and significantly lower salivary flow were found in children with CD [79]. 

Kuklik’s study [83] also aimed to analyze the incidence of MIH in patients with CD compared with the CG. Among participants with CD, 20 percent had MIH, while among those without the disease, only 5 percent showed the condition. This difference was statistically significant (*p* = 0.044), indicating an association between CD and the occurrence of MIH. Most of the defects in patients with CD consisted of demarcated opacities. All participants with MIH among those with CD had the classic form of the disease, characterized by gastrointestinal symptoms [83].

These studies suggest that CD is associated with an increased risk of MIH. Therefore, the early recognition of MIH could play an important role in the early diagnosis of CD. The regular dental management of celiac patients is recommended to prevent or manage any MIH-related complications. Further research is needed to confirm and further investigate these associations [79,83]. 

Khalaf’s research [82] involves adults with controlled CD and a CG without the disease. The results show that there are no significant differences in caries experience between the two groups, but the controlled celiac patients have fewer missing teeth than the CG [82]. The research also includes salivary assessments such as stimulated and unstimulated salivary flow, saliva buffering capacity, and bacterial counts of lactobacilli and streptococci mutans [82]. However, no significant differences emerged between celiac patients and the CG for these salivary measurements. In conclusion, while CD has been associated with various oral disorders, including enamel defects, this specific research shows no significant differences in the experience of caries between celiac patients and the CG [82].

Bramanti’s [90] work involved a total of 125 children, including 50 with established CD (Group A), 21 with suspected CD (Group B), and 54 healthy children (Group C). The investigation aimed to assess the prevalence of oral manifestations in patients with potential non-atrophic CD by comparing them with patients with established CD and healthy children [90]. The oral hard tissue abnormalities found were as follows: delayed tooth eruption (38% in Group A, 42.8% in Group B, 11.1% in Group C) and specific enamel defects (SEDs) (48% in Group A, 19% in Group B, absent in Group C). The presence of SEDs in patients with established CD was significantly higher than in patients with potential CD (48% vs. 19%, *p* = 0.0328). SEDs appear to correlate with histopathological lesions of the duodenum and atrophic villous lesions [90]. 

Other studies, also analyzing dietary habits and oral hygiene behaviors, point to a higher incidence of caries in patients with CD, with a significant impact on dental health [38,89,91]. 

Bulut’s study [38] group included 78 children, of whom 52 were previously diagnosed patients with CD and 26 were newly diagnosed children. A total of 100% of the previously diagnosed patients preferred fluoride-containing toothpaste, while in the newly diagnosed group, 26.9% preferred fluoride-free toothpaste [38]. No significant differences in dietary habits were found between the two groups [38]. Patients previously diagnosed with CD showed a significantly higher DMFT index than newly diagnosed patients. The frequency of visits to the dentist, preferences for toothpastes, and dental treatments were also significantly different between the two groups. However, there were no substantial differences in dietary habits and salivary parameters [38]. These results suggest that CD may affect dental health, with a higher incidence of caries in previously diagnosed patients. Collaboration between pediatric dentists and gastroenterologists is crucial for the effective monitoring and management of CD in children [38].

In De Carvalho’s study [89] of Brazilian children with a confirmed diagnosis of CD, significant findings emerged regarding the effects of the disease on dental hard tissue, particularly dental enamel. The research involved 52 children with CD and a CG of the same age and sex [89]. It was found that 61.54% of children with CD had defects in dental enamel, mainly systemic and classified as type I defects according to Aine’s classification [3]. This figure is significantly higher than the 21.15% found in the CG. In addition, a statistically significant association was observed between the specificity of DED and the presence of CD [89]. In detail, defects in enamel were predominantly concentrated in the incisors and molars, with canines found to be five times more affected in children with CD [89]. Chemically, the analysis of the composition of calcium/phosphorus (Ca/P) supply in the enamel of primary teeth revealed a significant decrease in the Ca/P ratio in children with CD compared with the CG. While specific elements such as oxygen (O), calcium (Ca), and phosphorus (P) showed no significant differences between groups, the lower Ca/P ratio in children with CD suggests possible changes in enamel solubility [89]. 

Shteyer’s study [91] included three groups of children: a group newly diagnosed with CD, a group of celiac children treated with a GFD for at least 6 months, and a CG of healthy children. Data were collected through clinical examinations, saliva analysis, and questionnaires regarding eating habits and oral hygiene behaviors.

Results indicated that 61.54% of children with CD had defects in dental enamel, particularly in the type I defect type. The prevalence of defects in enamel is significantly higher than the 21.15% found in the CG. In addition, a significant association emerged between the presence of CD and the specificity of DED, concentrated mainly in the incisors and molars. The analysis of enamel composition revealed a significant decrease in the calcium-to-phosphorus (Ca/P) ratio in children with CD [91].

From an oral hygiene perspective, the group of children with CD treated with GFD showed lower levels of plaque than the other groups, suggesting better oral hygiene in this group. The presence of defects in enamel was associated with a higher DMFT index, but no significant correlations were found between caries indices and the presence of bacteria in saliva [91]. 

Overall, these results suggest that CD can significantly affect dental health, with manifestations evident in dental hard tissues. However, while children with CD treated with GFD show improved oral hygiene, newly diagnosed children have an increased risk of caries [91]. These data underscore the importance of involving dentists in the management and awareness of CD, especially in newly diagnosed patients. Further large-scale prospective studies are needed to standardize oral hygiene awareness in newly diagnosed patients with CD [91].

Coelho’s study [40] of pediatric celiac patients in Portugal revealed significant data regarding oral health and dental hard tissue. Of the 146 participants, with a mean age of 10.5 years, 87.0% reported a history of at least one oral manifestation related to CD. The most common oral manifestations included recurrent aphthous stomatitis (RAS) (46.6%) and dental caries (45.2%). In addition, 32.4% reported improvements in oral health with the adoption of a GFD. Regarding oral health-related behaviors, 75.3% of parents said their children brush their teeth at least twice a day [40]. 

The increasing number of oral manifestations was found to be directly proportional to a deterioration in quality of life related to oral health (OHRQoL), underscoring the importance of identifying such manifestations early to improve the management and quality of life of pediatric celiac patients [40].

Villemur Moreau’s study [81] focused on the analysis of 28 children with CD (CD) and a CG of 59 children without CD. The mean age of children with CD was 8 years, with a higher prevalence in females (67.86%). The most common oral manifestations in children with CD included enamel defects (67.86%) and RAS (50%). In the CG, the prevalence of enamel defects was significantly lower (33.9%) [81]. Specifically, in children with CD, enamel defects were more frequent in deciduous molars (85.71%), permanent molars (64.28%), and canines (39.29%). The prevalence of enamel defects in children with CD was significantly higher than in the CG (57.14% vs. 13.56%). Aine’s classification showed that enamel defects in the CD group were more severe than in the CG (*p* = 0.04). Statistical analysis showed significant differences in the prevalences of enamel defects between children with CD and the CG [81]. 

Zoumpoulakis [86] suggests an association between the severity of DEDs and clinical manifestations of CD. In the study, 45 children with CD and a CG of the same age and sex underwent extensive examinations. The diagnosis of CD was made following the criteria of the European Society of Pediatric Gastroenterology and Nutrition, while the CG was examined for asymptomatic cases of CD using a rapid immunochromatographic test [86]. The results revealed that 64.4 percent of the CD group had enamel defects, specifically systemic defects of grade I, II, and IV according to Aine’s classification [3]. This prevalence was significantly higher than 24.46% in the CG, underscoring an association between CD and DED. The distribution of these defects mainly affected the permanent first molars, incisors, and deciduous molars [86]. An interesting aspect emerged from the genetic correlation, with a lack of significant association between specific HLA haplotypes and the prevalence of systemic enamel defects [86]. However, a significant correlation was found between the severity of DEDs and the specific form of CD, suggesting that diagnosis and disease categorization may influence the severity of defects. In the context of dental hard tissues, it is important to note that the defects were predominantly located in the buccal surface, with a significant distribution in the area of the incisura and upper half [86]. These findings clearly indicate an association between CD and SED, underscoring the importance for dental professionals to be aware of such oral manifestations to allow for timely referral when CD is suspected [86]. 

These findings are also confirmed by Ludovichetti [80] and Cantekin [65], who highlight the importance of the early detection of defects to identify the presence of CD.

Ludovichetti’s study [80] involved 114 pediatric patients divided into three groups: 38 with CD, 38 without CD but with other gastrointestinal conditions (non-CD), and 38 healthy patients (control). During dental examinations, DED and oral soft tissue lesions were recorded and subsequently graded [80]. In the CG, more than 70 percent of patients had enamel without defects (grade 0), while in the CD group, although there was an increase in defects, only 10.5 percent showed defects classified as grade III according to Aine. Incisors and molars are the most affected teeth in the CD and CG [80]. Soft tissue lesions, with the exception of migrating exfoliative glossitis, are more common in the CD group. Statistical analysis reveals a significant positive association between CD and enamel defects, with an odds ratio of 3.32 for the comparison between CD and non-CD, and 5.32 for the comparison between CD and control [80]. These results underscore that patients with CD have a significantly higher likelihood of developing SED and indicate the importance of the early detection of such defects to identify the presence of CD, allowing early intervention to prevent serious consequences [80]. 

The Cantekin study [65] involves 50 children, including 25 with CD and 25 healthy (control), aiming to examine correlations between CD and DED. In the CD group, the prevalence of enamel defects (48%) was significantly higher than in the CG (16%). Mean DMFT (deciduous) score values show no significant differences between the two groups (CD: 3.25 ± 3.25, Control: 4.56 ± 2.87), but mean DMFT (permanent) scores are significantly higher in the CD group (3.75 ± 2.62) than in the control (1.83 ± 1.78) [65]. In addition, 44% of CD patients have RAS, while no cases are observed in the CG. Statistical analysis confirms that the difference in the prevalence of enamel defects between the two groups is significant (*p* = 0.01) [65]. The presence of symmetrical defects, especially in incisors, suggests a correlation between CD and enamel defects. RAS is more common in CD patients, indicating a potential predictive role for this condition in the early diagnosis of CD. In conclusion, this study provides numerical evidence on the relationship between CD and DED, emphasizing the importance of the dentist’s role in the early diagnosis of this condition [65].

Pereira Macho’s research [84] indicates a significantly higher prevalence of DED in patients with CD than in the CG.

This is further confirmed by Amato [87], who also highlights enamel hypoplasia as a frequent manifestation in patients with CD [87].

Pereira Macho’s investigation [84] involved a total of 80 children with CD and 80 controls, all between the ages of 6 and 18. The results of the clinical examination, conducted by a certified dentist, showed that 64.4 percent of the group with CD had enamel defects, particularly grade I and II according to Aine’s classification [3]. This percentage was significantly higher than the 24.46 percent in the CG. The distribution of defects was mainly concentrated on the permanent first molars, incisors, and deciduous molars [84]. Although no significant association was found between specific HLA haplotypes and the prevalence of systemic enamel defects, a correlation emerged between the severity of defects and the specific form of CD, suggesting that the diagnosis and categorization of the disease may influence the severity of defects [84]. Overall, the group with CD had a significantly higher percentage of symmetrical enamel defects in the upper and lower first molars, as well as in the first molars in general and the upper lateral and upper central incisors. The prevalence of defects in enamel was significantly higher in patients with CD than in the CG, both in the permanent and deciduous dentition [84]. The classification of enamel defects according to Aine’s scale [3] showed that, in both groups, grade I defects were the most common. However, the percentage of grade I defects was significantly higher in the group with CD, both in the permanent and deciduous dentition. In addition, grade II was found only in the group with CD. Defects in enamel were more frequent in the upper and lower first molars, as well as in all first molars. In addition, more frequent defects were shown in the upper lateral incisors and upper central incisors [84].

Amato’s study [87] included 49 patients with completed CD and a CG of 51 healthy volunteers. Enamel hypoplasia was observed in 14.3% of CD patients compared to 0% in controls. In addition, a high prevalence of nonspecific tooth wear was found in the CD group (18.3%) compared with controls (5.9%) [87].

## 5. Limitations

This scientific paper reviews the relationship between celiac disease (CD) and dental enamel defects (DED), highlighting significant insights but also acknowledging limitations. These include a reliance on retrospective studies which may suffer from recall bias and inaccuracies, a lack of consistency in sample sizes, patient demographics, and methodologies across studies and the absence of standardized diagnostic criteria for both CD and DED. These factors introduce variability and may affect the findings’ applicability to diverse populations. Despite offering a thorough examination of existing literature, this paper emphasizes the importance of the careful interpretation of results and suggests that future research should focus on standardizing diagnostic approaches, including diverse populations, and utilizing prospective studies to establish clearer causal links and strengthen the evidence base.

## 6. Conclusions

The study highlights that DEDs are notably prevalent among CD patients and exhibit a systematic pattern, often presenting symmetrically and across all quadrants of the permanent teeth, particularly affecting incisors and molars. Despite these findings, routine screening for CD in patients with enamel defects is not universally advocated for. However, Aine’s classification may serve as a valuable tool for dentists to suspect CD. The research suggests a potential shared genetic link or an association with CD-induced nutritional deficits that could underlie the relationship between DEDs and CD. A significant association between CD and Molar Incisor Hypomineralization (MIH) indicates a heightened MIH risk in CD patients.

The early detection of such oral manifestations could lead to prompt CD diagnosis, even in the absence of gastrointestinal signs, thereby improving patient outcomes and facilitating timely treatment, including the adoption of a gluten-free diet, which is essential for managing CD and averting further health issues. Regular dental assessments are crucial for monitoring and addressing CD-related DEDs.

Moreover, CD can profoundly affect oral health and the oral health-related quality of life, particularly in pediatric populations, highlighting the need for integrated care involving both dentists and gastroenterologists. However, it is important to consider the study’s limitations, such as the high risk of bias due to confounding in many studies and the variability in selecting reported results, which could influence the reliability of these conclusions. Despite these challenges, the current evidence underscores the intricate link between CD and dental health, advocating for further extensive prospective studies to solidify and expand our understanding of this relationship.

## Figures and Tables

**Table 1:** Descriptive summary of item selection.

Author (Year)	Type of Study	Sample Size	Age	Type and Gravity of the Defects	Type of Analysis Performed	Outcomes
Bulut et al. (2023) [38]	Clinical study	78 P	4 to 15 y.o.	Enamel defects as per Aine’s classification observed in 34.5% of patients (Grade 1 in most cases, Grade 2 in 3.8%).	OE DMFT Dental plaque DED Analysis of unstimulated salivary flow rate and PH value	One group had higher dental decay scores with no differences in salivary metrics, 34.5% had mild enamel defects, and aphthous stomatitis was more common in recently diagnosed individuals.
Coelho et al. (2023) [40]	Cross-sectional study	146 CD P	10.5 years	RAS, DC, and DO	Online questionnaire for parents Oral health behaviors History of oral manifestations	RAS (46.6%), DC (45.2%), and DO (39%) was the most reported oral manifestation among CD P.
Elbek-Cubukcu et al. (2022) [79]	Prospective cohort study	62 CD P, 64 CP	n.r.	MIH, RAS, poor oral hygiene, and dental status	OE DMFS DFS MIH assessment	Prevalence of DED: in the group with CD: 61% (MIH); in the control group 65.6% (no MIH).
Ludovichetti et al. (2022) [80]	Ob S	114 P	6–14 y.o.	DEDs, specifically hypoplasia; Severity classified by Aine’s classification (Grade 0, Grade I, Grade II/III)	OE Fischer’s Test	DEDs were present in 31.6% Grade 0, 34.2% Grade I, 23.7% Grade II/III in the celiac disease (CD) group. In the non-CD group, 60.5% had no defects, 31.6% Grade I, 7.9% Grade II/III. Control group mostly had no defects (71.1%).
Ahmed et al. (2021) [1]	Prospective CC study	118 P	20–37.23	DED observed in 66.9% of patients with CD, specific/bilaterally symmetrical more common in treatment-naïve and GFD-treated patients compared to controls (20%)	Statistical analysis using GraphPad Prism 8 software, with χ^2^ test or Fisher’s exact test	Significantly higher percentage of CD patients (68.6%) reported xerostomia/dry mouth sensation compared to controls (7.5%), DED 8.1 (95% CI 3.4–19.2), xerostomia 27 (95% CI 7.8–93.2).
Villemur Moreau et al. (2021) [81]	Ob S	28 CD and 59 CP	3–12 y.o.	DEDs graded according to Aine’s classification (grades I to IV)	OE	CD children had significantly more enamel defects and recurrent aphthous stomatitis than the control group (67.9% vs. 33.9% for ED, and 50.0% vs. 21.8% for RAS, respectively). No significant delay in dental eruption was observed in CD children. EDs in CD children were more severe than in the control group (*p* = 0.04).
Khalaf et al. (2020) [82]	CC	23 CD P, 23 CP	39.1 ± 14.4 years	Dental enamel hypoplasia, aphthous ulcers, dental caries (DMFT)	OE Rx evaluation Saliva test	Significant inverse relationship between MIH and age at diagnosis of celiac disease: Marsh 2 damage type in celiac disease is associated with an increased risk of dental caries. Reduced buffering capacity of saliva in children with celiac disease.
Kuklik et al. (2020) [83]	Prevalence study	40 CD P 40 CP	16.5 y.o.	MIH with demarcated opacities, post-eruptive breakdown (PEB), atypical restoration	OE of teeth under natural light using a flat mirror and a blunt tip	Out of 80 participants, 10 had MIH (12.5%). Among the 40 celiac patients, 8 had MIH (20%). Among the 40 individuals without CD, 2 had MIH (5%). Celiac disease increased the likelihood of MIH occurrence by 4.75 times compared to the CG.
Pereira Macho et al. (2020) [84]	CC	160 P	6–18 y.o.	Mainly symmetrical enamel defects characterized by pitting, grooving, and loss of enamel	OE	Grade I and Grade II defects were observed in both groups, but significantly higher in celiac group (*p* = 0.002, *p* = 0.003). Symmetric enamel defects were more prevalent in celiac group, particularly in first upper molars, first lower molars, lateral upper incisors, and central upper incisors (*p* < 0.05).
Shahraki et al. (2019) [85]	Prospective Ob S	65 CD P 60 CP	Ages 3–16 y.o.	DED symmetric and non-symmetric; Grades I–IV based on Aine’s criteria	OE	Half of the patients with celiac disease exhibited enamel defects, predominantly mild but including some severe cases, with a higher incidence of tooth decay in baby teeth, more frequent dry mouth symptoms, but not in adult teeth, despite reduced sugar intake compared to the control group.
Zoumpoulakis et al. (2019) [86]	Comparative, Cross-sectional	45 CD P, 45 CP	10.3 ± 4.1 y.o.	Systemic and non-systemic DED	OE DMFT	Prevalence of systemic DED was significantly higher in CD patients (51.1%) compared to controls (11.1%).
Amato et al. (2017) [87]	CC	49 CD P 51 CP	CD P: 31.8 ± 11.58 y.o., CP: 30.5 ± 8.7 y.o.	Enamel Hypoplasia: Aine Grade 1 (4 patients), Aine Grade 2 (3 patients); Non-specific Tooth Wear: Smith and Knight Index Grade 1 (4 patients), Grade 2 (3 patients), Grade 3 (2 patients)	Saliva Analysis, DMFT	RAS: CD Patients 53.0%, Controls 25.5%; Aphthosis during visit: CD Patients 0%, Controls 0%; Atrophic Glossitis: CD Patients 0%, Controls 0%; Enamel Hypoplasia: CD Patients 14.3%, Controls 0%; Non-specific Tooth Wear: CD Patients 18.3%, Controls 5.9%.
de Queiroz et al. (2017) [88]	Retrospective Ob S	45 CD p	Age range: 2–15 y.o.	DED Grades I-IV based on Aine’s criteria 55.6%	OE DEDs phenotype determination using Aine classification	DED prevalence 55.6%.
Cantekin et al. (2015) [65]	RS	25 CD P, 25 CP P	8.94 ± 2.08 (CD) and 9.66 ± 4.26 (CP) y.o.;	DED prevalence was higher in CD children (48%) than healthy children (16%). Enamel defects were generally symmetrical and mostly observed in anterior teeth.	DDMFT scores	DMFT scores were significantly higher in CD children (3.75 ± 2.62) compared to the control group (1.83 ± 1.78). RAS prevalence was higher in CD children (44%) compared to the control group (0%). Significant differences were found between CD and control groups for both enamel defects (*p* = 0.01) and DMFT scores (*p* < 0.01).
de Carvalho et al. (2015) [89]	CC	52 CD P and 52 CP; additional 50 DEDs	2 to 15 y.o.	DEDs graded according to Aine’s classification (grades I to IV)	DEDs RAS dental caries experience salivary parameter analysis of primary enamel molars using energy dispersive x-ray spectroscopy and Fourier transform infrared spectroscopy.	Children with celiac disease had more dental enamel defects and mouth ulcers but fewer cavities, showed signs of altered enamel chemistry with lower salivary flow and altered calcium-to-phosphorus ratios, although their carbonate-to-phosphate ratios were comparable to those of a control group.
Bramanti et al. (2014) [90]	Prospective Cohort	116 PP	2–16 years old	Specific Enamel Defects (SED)—Grades I-IV (severity) Unspecific Enamel Defects (UnSED) Dental Caries (DMFT/dmft indices) Dental Delayed Eruption (DDE)	Cross-Sectional Study	Anomalies found in oral hard tissues: Delay in tooth eruption: ○ Group A: 38% ○ Group B: 42.8% ○ Group C: 11.1% Specific enamel defects (SED): ○ Group A: 48% ○ Group B: 19% ○ Group C: Absent The presence of SEDs in patients with established celiac disease is significantly higher than in patients with potential celiac disease (48% vs. 19%, *p* = 0.0328).
Shteyer et al. (2013) [91]	PS	90 P, 30 in each group (newly diagnosed CD, CD treated with Gluten Free Diet, and control).	1.4 to 18 years;	DEDs graded according to Aine’s classification (grades I to IV).	OE Saliva sampling for bacterial and pH analysis DMFT/dmft index Plaque index.	Higher prevalence of enamel hypoplasia in CD children (66%).
Trotta et al. (2013) [48]	Prospective Ob S	54 P	37 ± 13 years	DED observed in 85.2% of CD P, predominantly Aine grade 1 type lesion (33.3%)	DED according to Aine (Table 1) Chi square test for association with clinical type and age at diagnosis	Severe DED (Aine grade 3 and 4) more common in classical CD (10/32) than non-classical CD (2/20), not statistically significant.

## Data Availability

Not applicable.

## Abbreviations

CP	Control patient
CC	Case control
CD	Celiac disease
CG	Control group
DC	Dental caries
DED	Dental enamel defects
DMFT	Decayed, missing, and filled teeth
DFS	Decayed, filled tooth surfaces
DO	Dental opacity
EAPD	European Academy of Pediatric Dentistry
ECOHIS	Early Childhood Oral Health Impact Scale
GFD	Gluten-free diet
MIH	Molar incisor hypomineralization
Ob S	Observational study
OE	Oral examination
OHRQoL	Oral health related to quality of life
PEB	Post-eruptive breakdown
PS	Preventive study
RAS	Recurrent aphthous stomatitis
SED	Specific enamel diseases
(TTG) IgA	Tissue transglutaminase antibodies
